# ‘I might be lucky and go back to school’: Factors affecting inclusion in education for children with disabilities in rural Malawi

**DOI:** 10.4102/ajod.v11i0.981

**Published:** 2022-11-14

**Authors:** Lena M. Banks, Xanthe Hunt, Khumbo Kalua, Providence Nindi, Maria Zuurmond, Tom Shakespeare

**Affiliations:** 1 International Centre for Evidence in Disability, London School of Hygiene & Tropical Medicine, London, United Kingdom; 2 Department of Global Health, Faculty of Medicine and Health Sciences, Institute for Life Course Health Research, Stellenbosch University, Bellville, South Africa; 3 Lions Sight First Eye Hospital, Blantyre Institute for Community Outreach (BICO), Blantyre, Malawi

**Keywords:** inclusive education, Malawi, exclusion, disability, school

## Abstract

**Background:**

Globally, children with disabilities are often excluded from and within schools.

**Objectives:**

This study explored experiences of inclusion in education amongst children with disabilities in Malawi. The enquiry focused on the perspectives of children and their caregivers on barriers and enablers of inclusion.

**Method:**

Data were gathered through in-depth interviews with 37 children with disabilities, 61 caregivers and 13 teachers from Ntcheu and Mangochi districts and analysed thematically using the International Classification of Functioning, Disability and Health as a framework.

**Results:**

Overall, this research study found that children with disabilities face persistent and systemic barriers to attending, progressing and learning in school.

**Conclusion:**

School outcomes were influenced by a range of impairment-related, personal and environmental factors, including poor health, household poverty, attitudes of caregivers, teachers, peers and children themselves and school resources for inclusive education.

**Contribution:**

These findings carry implications for policy and planning in inclusive education and other services to support the health and well-being of children with disabilities in Malawi.

## Background

Universal access to quality education is a human right. International consensus documents, including the Sustainable Development Goals (SDGs), codify this right and provide clear targets for countries to try and achieve in terms of educational access (United Nations 2015). Universal access to quality education is also essential to poverty reduction.

The right of children with disabilities to education is codified in Article 24 of the United Nations Convention on the Rights of Persons with Disabilities (UNCRPD) (United Nations 2006), and the SDGs (particularly Goal 4, to ensure inclusive and equitable quality education and promote lifelong learning opportunities for all) recognise the importance of ensuring accessible education (United Nations 2015).

However, the world’s 240 million children with disabilities (United Nations International Children’s Emergency Fund [UNICEF] 2021) face a range of barriers that limit their access to and participation in education. Exclusion from the educational environment and from educational attainment is still persistent, particularly in low- and middle-income countries (LMICs), despite some gains made in the past few decades (Mizunoya, Mitra & Yamasaki 2018; UNICEF 2021). Compared with their peers without disabilities, children with disabilities are less likely to enrol in school, and if they do enrol, they have lower levels of school attendance and lower rates of transition to higher education than their peers without disabilities (Banks et al. 2017; United Nations 2019; World Health Organization [WHO] & World Bank 2011). A landmark study in 2018 showed that across 15 LMICs, having a disability reduced the probability of a young person attending school by 30.9% (Mizunoya et al. 2018).

A variety of challenges can create barriers to educational inclusion, participation and attainment amongst children with disabilities, including inaccessible school facilities and transport to schools, inadequate teacher skills in inclusive education, caregiver and teacher attitudes and lack of resources to support inclusive education (Banks et al. 2019; Singal et al. 2015; Taneja-Johansson, Singal & Samson 2021; United Nations 2019).

A recent analysis by Le Fanu, Schmidt and Virendrakumar (2022) puts forward a useful conceptualisation of inclusive education based on General Comment 4 on Article 24 of the CRPD, to which this article adheres. These authors hold that inclusive education can be conceptualised as having the following dimensions: *longitudinal* (it should be lifelong), *location* (it should be available to children near to where they live), *pedagogical* (it should include quality learning opportunities), *environmental* (it should include efforts towards social inclusiveness and physically accessibility in schools) and *consequential* (the results of inclusive education should be visible in educational and social outcomes amongst children with disabilities).

Inclusive education, as articulated in General Comment 4 on Article 24, includes reasonable accommodations, continuous personalised support, access to needed assistive technologies and adapted curricula. Both of these strategies entail the use of contextually appropriate teaching and learning adaptations, which are responsive to the needs of children with disabilities in the classroom (Le Fanu et al. 2022).

Some of these challenges and barriers may be more significant in some settings than others, making it important to understand contextual variation in experiences. By isolating the factors limiting participation in a specific setting, it may be more possible to identify the best ways of promoting inclusion of children with disabilities in education.

This research study explores barriers and enablers to inclusion in education for children with disabilities in Malawi, with a focus on the perspectives of children and their caregivers.^1^

### Education and disability in Malawi

Estimates on the prevalence of disability amongst school-aged children in Malawi vary from 0.43% to 5.60% (Mizunoya et al. 2018; UNICEF Malawi 2020). The Ministry of Education, Science and Technology (MoEST) is responsible for formal education in the country, including for children with disabilities (Deputy Director [District Education Office Ntcheu] pers. comm., 2015). By law, primary education is free in Malawi. However, secondary schools may charge school fees and often have a limited number of spaces available.

The main model for inclusive education in Malawi is resource classrooms, special education units within mainstream schools where children with disabilities receive specialised instruction and extra resources to support their learning. As of 2020, there were 60 and 88 resource rooms at the primary and secondary level, respectively (Ministry of Education Malawi [MoEM] 2021) – a small fraction of total classrooms (0.8% for primary and 1.3% of secondary) (MoEM 2021). There are also some so-called ‘special schools’ that provide instruction to children with disabilities in segregated settings. These schools are primarily for children with vision and hearing impairments. However, reflective of the shift from segregated to inclusive education, many of these schools are being converted into resource centres (Artiles et al. 2015). Still, data from 2016 to 2017 indicate that the vast majority – 98% of primary school students and 93% of secondary students with disabilities – attend mainstream schools, where inclusive education resources are unlikely to be provided (UNICEF Malawi 2020).

Malawi is signatory to several international conventions that outline the rights of children with disabilities to education (Artiles et al. 2015). Moreover, the priorities enshrined in these conventions are codified and in some instances operationalised in Malawian laws and policies, such as the *Disability Act* (2012). The country’s commitment to equal access to and inclusion in education for children with disabilities is reflected in the National Policy Guidelines on Special Needs Education (2007), the National Education Investment Plan 2020–2030, the National Disability Mainstreaming Strategy and Implementation Plan 2018–2023 and the National Policy on the Equalisation of Opportunities for Persons with Disabilities (2006) (Eide & Munthali 2017; UNICEF Malawi 2020).

Additionally, the Malawi Growth and Development Strategy III 2017–2022 includes several disability-specific education goals (Government of Malawi 2017), and Malawi has a National Strategy on Inclusive Education (2017–2021), which covers eight priority areas, including improving capacity for inclusive education (e.g. teacher training, school resources and school accessibility improvements), learner identification and needs assessments and increased funding for and monitoring of inclusive education roll-out (Banks & Zuurmond 2015).

Even though there is a strong legislative basis for inclusion in education, gaps in implementation remain. A national survey in 2012–2013 found 44% of primary school-aged children with disabilities were out of school compared with 13.2% of those without disabilities (gap of 30.8 pp, *p* < 0.001), which widened even further for secondary school-aged students (68% vs. 21.6% non-attendance, gap of 46.5 pp, *p* < 0.001; Mizunoya et al. 2018). These numbers may have improved since this survey: the Ministry of Education collects data on enrolment of children they identify as having special education needs, and there has been more than a doubling of enrolment in primary school (from 83 666 in 2009 to 186 501 in 2020) and quadrupling of secondary school enrolment (2780 in 2009 to 10 290 in 2020; MoEM 2021; UNICEF Malawi 2020).

According to the 2018 Malawi Population and Housing Census estimates, there are approximately 333 000 children of school-going age with disabilities nationwide. For the 2019–2020 school year, the Ministry of Education had identified 196 and 791 primary and secondary school students, respectively, with special education needs (Government of Malawi 2020), and thus it is unclear if the remainder are still out of school or not counted in official records as having a disability.

Two past studies by De Souza (2021) and Chirwa, Lingolwe and Naidoo (2021) have explored perceptions and experiences of inclusive education amongst teachers in Malawi. Both studies found that the implementation of inclusive education in the country has been marked by challenges, in part stemming from a lack of orientation and training amongst the teachers tasked with transforming their mainstream classrooms into inclusive ones and in part a result of a lack of resources to support inclusive education (Chirwa et al. 2021, De Souza 2021).

However, little qualitative research has been carried out in respect of schooling for children with disabilities and their caregivers in the country, and therefore little is known about their lived experiences of school and its educational and social dimensions.

In this study, the International Classification of Functioning, Disability and Health (ICF) is used as a framework for thinking about experiences of education amongst children and their caregivers and teachers in Malawi. The ICF includes attention to the following dimensions:

- body functions and structures of people and impairments of body functions and structures
- activities and their limitations
- participation and its restrictions
- environmental factors.

Under the ICF (and indeed the UNCRPD), the extent to which an impairment leads to participation restrictions is influenced by the interaction between an individual’s impairment and personal and environmental factors. Importantly, the ICF is also a biopsychosocial model of disability and thus necessitates thinking about individuals with disabilities as embedded in families, communities and countries. As such, the interview schedule, the way the data was analysed and the manner in which it is presented below pays attention to interactions of children’s impairments and the environment at the individual, family, school and community level, as well as how these result in participation restrictions.

## Methods

This study uses a qualitative design involving in-depth interviews with children, their caregivers and teachers. A qualitative methodology was deemed to be appropriate, as an understanding of the experiences of education was sought from key stakeholders’ own perspectives. In-depth interviews were selected because it was desirable to provide space for each child, caregiver and teacher to provide their own account and understanding of the phenomena; it was also important to ensure that tailored accommodations could be made for each child based on his or her specific needs.

All participants were recruited from population-based surveys conducted in two districts, Ntcheu (Central region) and Mangochi (Southern region). The 53 participants from the Ntcheu district were recruited as part of the Key Informant Method (KIM) Child Disability Project (for full study details, see Tataryn et al. 2017). The 58 participants from Mangochi were recruited for the DeWorm 3 study (Ásbjörnsdóttir et al. 2018). Based on the surveys, the prevalence rate of childhood disability (ages 0–18) was found to be 1.7% in Ntcheu and 3.7% in Mangochi (Tataryn et al. 2017).

In both the settings, children were purposively recruited using demographic data from the underlying surveys to ensure representativeness by type of impairment or functional limitation, gender and school status (in vs. out of school) (see Table 1 for details). In Ntcheu, children of 12–18 years were eligible for inclusion, as this study was focused on children transitioning to secondary school. In Mangochi, selected children were 6–14 years old, as the underlying study focused on mass drug administration for soil transmitted helminths delivered to primary school children. The two studies were conducted separately with different research teams and some differences in aims, which explains the differences in the study sample. However, both used similar interview guides to collect data.

**TABLE 1:** Participant demographic details.

Variables	Ntcheu	Mangochi
**Sample size interviewed**		
Caregivers	23	38
Children	17	20
Teachers	13	0
**Details of selected children** †		
Age range (years)	12–18	6–14.5
**Gender**		
Male	11	20
Female	12	18
**Impairment type** ‡		
Visual	4	10
Hearing	7	10
Intellectual	9	21
Physical	9	13
Epilepsy	3	0
Albinism	1	0

† , Details of children from both caregiver and child interviews;

‡ , Not mutually exclusive categories.

Interviews were conducted with children and their caregivers in both sites, and in Ntcheu district, teachers of children with disabilities were included. In Ntcheu, data collection was undertaken in October – November 2015, whilst in Mangochi, data were collected in March 2020. Teacher interviews were planned but were unable to proceed in Mangochi because of the commencement of the coronavirus disease 2019 (COVID-19) pandemic and the closure of schools.

A semistructured interview guide was used, with children and their caregivers interviewed separately. Details on the child’s communication needs were sought in advance. Interviews were conducted in Chichewa or Yao. In Ntcheu, interviews were conducted by L.M.B., a non-Malawian, female researcher with translation support. In Mangochi, interviews were conducted by local data collectors with supervision from P.N. All of the interviews were audio-recorded, transcribed and translated into English for analysis by trained transcribers and translators. Interviewers also took detailed notes during the interviews, and these were shared with the analysis team to support framing of the transcript data.

The topics that were covered in the caregiver interviews included: (1) family background; (2) the child’s impairment, abilities and general health, including access to health or rehabilitative services; and (3) the child’s education, including social and academic experience in school and/or reasons for non-attendance. For the child interviews, the interviewers used a visual tool to prompt discussions about schooling. The interviewers provided children with emotion cards (faces with ‘Happy’, ‘Sad’ and ‘Angry’ expressions) and asked about their experiences at home, on the way to school, in the classroom, in the playground and in using the toilet facilities. Children were prompted to use the cards to indicate in which settings they felt which emotions, and then these links were explored. Teacher interviews in Ntcheu focused on their observations of the sampled child’s experience in the school. They were also asked some broader questions about their own experiences and reflections on teaching children with disabilities.

After each day of fieldwork, interview notes were reviewed by the lead field researchers (L.M.B. in Ntcheu, P.N. and X.H. in Mangochi) and the interviewers. Data coding was managed using NVivo 10, a software for qualitative data analysis. Thematic analysis was used to analyse the data, with independent coders examining the transcripts to identify units of meaning, synthesising these units, where necessary, into larger concepts maps (themes), and then examining the inter-relationships between themes and different participant characteristics (e.g. gender and impairment type). Given that the ICF, reinforced by the UNCRPD, was used as a framework to guide the researchers’ engagement with the data, themes were ultimately organised in a manner, which corresponded to the ICF’s framing of disability and functioning.

### Ethical considerations

Ethical approval for each of the studies was received from the London School of Hygiene & Tropical Medicine’s Observational / Interventions Research Ethics Committee (ref. no. 6409‑01 and 17637) and the University of Malawi’s College of Medicine Research and Ethics Committee prior to commencing data collection. Before the start of each interview, informed written consent was received from participants above the age of 16 years. For younger children and those with communication or intellectual impairments, a simplified oral assent was sought, and pictorial child-friendly information sheets were developed. Referrals for health and child protection services were provided as needed. The study was conducted in accordance with the Helsinki Declaration as revised in 2013.

## Results

Data from 61 children (23 from Ntcheu; 38 from Mangochi) and 13 teachers (all Ntcheu) were collected (Table 1). For the 61 children, data were gathered through 61 caregiver interviews (23 from Ntcheu; 38 Mangochi) and 37 child interviews (17 from Ntcheu; 20 from Mangochi). Non-response in children was because of severe communication difficulties (e.g. deaf with no knowledge of a formal sign language, severe intellectual impairment).

Table 2 describes the main themes and subthemes from the thematic analysis. However, it is worth noting that often the factors that affect each child’s access to and experiences of school are complex and interrelated. For instance, the deprivations associated with poverty could keep children at home working instead of in schools, but household poverty could also be worsened by the costs associated with disability. As such, child absenteeism from the school could not be seen as purely because of the child’s impairment nor purely because of the economic circumstances of the household; rather, it is because of a dynamic interaction between them.

**TABLE 2:** Themes and subthemes.

Component of the ICF	Subtheme
Health and impairment-related factors	Poor health and access to health services
Personal factors	Household poverty
	Child’s motivation
Environmental factors	Social attitudes
	Resources for inclusive education
	Journey to school
Impact of non-inclusive education on participation	Poor learning outcomes
	Social exclusion and isolation

ICF, International Classification of Functioning, Disability and Health.

### Health and impairment-related factors

#### Poor health and access to health services

Across both settings, participants observed that poor health amongst students with disabilities, as well as the need for treatment, was a significant reason for absenteeism, difficulties learning, grade repetition and, in a few cases, non-attendance. Often, health problems in children were related to their impairment or health conditions. Several children had intellectual and physical impairments stemming from unmanaged epilepsy or sensory impairments from eye and ear infections. The children’s impairments were the result of health conditions going untreated for a long time. At the time of interviewing, the children had long-term impairments, and many experienced frequent flare-ups of the underlying health condition. In some cases, new episodes caused pain and worsening severity of impairment:

‘I sometimes fail in class because I can’t see what’s on the board, and I also miss classes when I have the [*eye*] swellings while my friends are learning. [*How often is it that you can’t see the board even when you sit at the front?*] It’s not all days; it’s only when my eyes are itchy and tears come out that I can’t see what’s written on the board, and that is why I fail. [*How often does that happen in a month?*] Three days a month … It happens when my medication is finished.’ (girl, age 15, visually impaired, in school)

Participants also observed that seeking health care and treatment for their impairment or impairment-related symptoms led to frequent absences from schools. Distance was a particular challenge for rehabilitation and other disability-related specialist services, which tended to be far from where people lived. Several children were kept away from school – in some cases starting school late or failing to attend altogether – because the caregivers were seeking health care for them, including potential ‘cures’. In these instances, some caregivers thought that their child could not be educated without the resolution of his or her impairment.

In a few instances, the involvement of community-based organisations was helpful for accessing needed health and rehabilitation services. Even receiving an expert opinion on realistic expectations for their child’s disability in a few cases was helpful at preventing caregivers from spending unnecessary time and money searching for cures or unneeded treatments.

## Personal factors

### Household poverty

Financial hardship and poverty were dominant themes that were present in almost all interviews. Even though primary school is free in Malawi, there are still some costs associated with schooling, for which caregivers are financially responsible. These costs include yearly registration, uniforms and school supplies. Given the high level of poverty in the sites, these costs (although small) were unmanageable for families. Although many households in the study settings were living in poverty, the research found indications that households with children with disabilities may have been particularly affected. Many caregivers reported spending additional time caring or seeking services for their child with a disability, which could reduce the time spent on economically productive activities. Most households with children with disabilities reported additional disability-related costs, particularly for health services, which reduced their capacity to pay for other expenses, including for schooling.

Poverty was a dominant factor across both settings for children being out of school, missing classes or having a range of other difficulties with learning. One of the children (girl, age 12, hearing impaired, not in school) explained how shame and bullying over her family’s financial situation deterred her from going to school:

Interviewer ‘What would make you go back to school?’

Girl ‘If I had somewhere to write, a pen and a pencil.’

Interviewer ‘When you were going to school, didn’t you have those materials?’

Girl ‘I didn’t have any … we were writing on the floor.’

Interviewer ‘What else would make you go to school?’

Girl ‘A uniform and a dress.’

Interviewer ‘Do the other kids at school also wear uniforms?’

Girl ‘Yes …’

Interviewer ‘What do your friends say about you not going to school?’

Girl ‘They tell me to go to school.’

Interviewer ‘And what do you say?’

Girl ‘I tell them I don’t have clothes to wear to school … Other kids would be laughing at me when I wore dirty clothes.’

In Malawi, secondary schools may charge a fee, and in some cases, this is prohibitive for families, preventing children with and without disabilities from progressing. Many secondary schools are concentrated in urban areas and far from where children live, resulting in further costs for accommodation and travel. Almost all families highlighted costs as the main reason why education beyond primary school was unlikely for their child.

Perceived cost was also the primary reason which caregivers provided for not sending their children to special schools. One of the fathers explained that although his 17-year-old son had been accepted to a special school that was based several districts away, he ‘failed to support transport because of my poverty condition’. Other caregivers reported that they had not investigated special or resource schools as they assumed the costs would be too high.

Just under a third of children included in this study had missed their school to work, mainly in the home. This was experienced as unfair by some children, as exemplified by one child’s (boy, age 13, epileptic, in school) reflection on being kept home to work:

Interviewer ‘What makes you angry at home?’

Boy ‘If my mother tells me not to go to school … I want to go to school.’

Interviewer ‘Why does she tell you not to go to school?’

Boy ‘She says I should look after the baby when she goes to the fields.’

### Motivation

The majority of children and their caregivers held positive attitudes towards education. As a mother of a boy aged 15 with epilepsy and profound hearing and intellectual impairment who is not in school explained, the key reasons why her son and other children wanted to attend school were because they wanted to learn and spend time with their peers:

‘He started [*school*] on his own, he would admire his friends. He followed his friends to school, then he demanded that we buy a notebook for him … He just wants to learn … When he was at home he would scribble on the floor. You could see that if he was alright, he could have been educated.’ (boy, age 15, epileptic and multiple impairments, not in school)

Some children who had dropped out of school expressed a keen desire to return. An 18-year-old boy explained that even though he was top of his class academically in primary school and was accepted into a secondary school, he was unable to attend because of school fees. Still, he had kept his old notebooks and reviewed them frequently to ‘remind myself what I learned in class, because I might be lucky and go back to school’.

## Environmental factors

### Social attitudes

#### Caregivers

Many caregivers were supportive of their child pursuing an education, endorsing the idea that education was the gateway to a better future to ‘gain knowledge’ and ‘a better job’. Some reflected that their own socio-economic problems were because of a lack of education, and thus they wanted a different future for their children. Similarly, others felt that the types of jobs that were common in their community (mostly in agriculture, involving manual labour) would be difficult for their children to perform, particularly for children with mobility limitations, and they hoped education could lead to other desk-based jobs.

Still, several caregivers interviewed were not sure of the use of sending their children to school and questioned the ability of their children to learn. These attitudes were influenced in part by the lack of resources for inclusive education at local schools. The mother of a 16-year-old girl with a profound visual impairment explained why her daughter had dropped out:

Mother ‘From the way I look at it, I don’t think she can manage, because she doesn’t see what has been written on the board … I felt that since she doesn’t see properly, then she can’t continue with school; she also said she wants school but since she is not able to see, she just accepted that she will just be staying at home.’

Interviewer ‘How does [*child*] feel now that she doesn’t go to school?’

Mother ‘She doesn’t feel good about it; she sometimes cries that had it been that she continued schooling she would have been in Form 1.’

Others were unaware of their child’s right to an education or were unsure how to advocate on behalf of their children within the school system. Several caregivers felt that sending their child to school would burden teachers and peers. This was particularly the case where children had behavioural challenges. An 18-year-old girl with intellectual and physical impairments had never been to school, as her mother explained that ‘it will be difficult for the teacher to teach other children, she will give her a burden’.

These opinions were in some cases reinforced by actions from teachers and school staff, who had suggested or explicitly requested that children not be sent to school. Some caregivers were hesitant to request accommodations that could assist with their child’s learning. The mother of a 13-year-old child with a hearing impairment explained that she had not spoken to teachers about giving her daughter a front seat, as she was afraid ‘I would look like I am troubling them by telling them what to do’. Still, a few others took proactive roles, taking time to meet their child’s teachers to explain their child’s impairment and small accommodations that might help their learning.

Concerns for their child’s safety was a common reason for keeping children, particularly those with high support needs, out of school. The mother of a 13-year-old boy, who has multiple impairments, explained that they stopped him from going to school when he tried to follow his friends, as ‘[*teachers and peers*] beat him up. I have heard that they beat him at school. That’s when we follow him and stop him from going to school’. Fears of children, particularly with intellectual or visual impairments, getting lost on the way to school were also reported in a few instances. Safety was a concern, particularly for girls, when attending schools far away, which is common for secondary schools. These safety concerns should also be viewed within the context of many children and caregivers reporting discrimination and abuse, both at school and in the community.

#### Teachers

Attitudes of teachers towards children with disabilities were mixed. Some children reported positive relationships with their teachers, including ones who had taken additional steps to support their learning or personal development. A girl with a visual impairment explained that ‘the teachers gave me a front seat and write big fonts, and [*I*] am able to see’. Similarly, the grandmother of a child with a hearing impairment explained that a teacher had bought clothes and food for her child. However, in several instances, teachers or school staff had asked caregivers not to enrol or stop their child from coming to the school. For instance, the caregivers of a 17-year-old girl with multiple disabilities reported that teachers at the local school had refused to enrol her:

Mother ‘[*W*]hen the teacher saw her condition, they said she couldn’t start school but they told us that we should go with her to the hospital …’

Father ‘When she went with her mother to enrol her, they rejected her. [*Then*] I went there. They said the child needs another school. At that time, we didn’t know of the other school.’

Concerns over disruptive behaviour were a major reason provided for why teachers requested students not to attend, whilst a few felt that the child should prioritise receiving medical care before coming to the school. In speaking to teachers in Ntcheu, some questioned the place of children with disabilities in mainstream schools. Several felt that children with disabilities would be better served in special schools, which they believed had the resources and the mandate to teach such children. Interestingly, these attitudes were held even with respect to a few of their students with moderate impairments, who they reported were doing well academically.

#### Peers

Almost all children faced victimisation at school, including bullying, discrimination and other forms of violence. In most cases, classmates were the perpetrators of the abuse. In some cases, fear of victimisation affected children’s desire to go to school. The mother of a 14-year-old boy with physical and intellectual impairments explained that:

‘[*His classmates*] tease him that he is disabled; they also beat him … and steal his food … In the past he used to run away from school … he would sometimes say he will stop school, but I encourage him.’ (boy, age 14, multiple impairments, in school)

Similarly, the mother of a 13-year-old girl with intellectual and physical impairments discussed the impact of bullying:

‘I just hear from her friends that [*my child*] did something bad [*at school*] but I know that it’s because the others are not used to her … they have problems communicating … because of her mental status, she sometimes annoys her friends and they beat her … [*and*] make fun of her arm condition and her dumb condition … It affects her … sometimes she just stays quiet thinking about it.’ (girl, age 13, multiple impairments, in school)

Still, positive attitudes of peers were an important enabler to attending school or learning. Some children with sensory impairments reported that friends lent them notes if they could not see the board or hear the teacher’s instructions. A few children pointed to friendships with their peers as helping them feel less isolated in the face of bullying from other classmates. For example, a 16-year-old boy with a physical impairment explained:

‘I love my friends. They don’t gossip about me and aren’t violent. But there are other learners who keep saying that they can’t be friends with me because of the way I walk … [*My friends*] just tell me to leave them alone and that maybe their whole family does not have people with disabilities … [*so*] I just tell [*the kids who tease me*] that God should bless them, I just walk with the ones that like me….’ (boy, age 16, physical impairment, in school)

### Resources for inclusive education

All categories of participant in this study – children, teachers and caregivers – suggested that schools are often not adequately equipped to include and accommodate students with disabilities, particularly children with more complex learning needs such as children with intellectual impairments or profound hearing and visual impairments. As one of the mothers explained, the lack of accommodations and support for her child led to limited learning whilst in the classroom:

‘[*His hearing impairment*] affects his life more especially when it comes to his educational side of it. His classmates are able to hear when they have learned in class, but he cannot; when he comes home and we ask him what he has learned, he tells us parallel things. Hence, we are worried because our child is not able to hear in class.’ (mother of a boy [age 8, hearing impairment, in school])

Several caregivers and children reported that they only needed minor adaptations, such as being placed at the front of the classroom to better hear the teacher or see the blackboard. Sometimes these minor adaptations were provided, but in other cases they were denied. A caregiver of a child with a hearing impairment explained that she had asked for him to sit at the front but was told that he was too tall, which would block the view of other children.

Teachers, caregivers and children alike observed that large class sizes were a major barrier to learning and teaching. Teachers in mainstream schools in Ntcheu who were interviewed reported class sizes of 100–186 students, which was double to triple the maximum class size of 60, which was in place at the time of data collection. The high ratio of students to teacher made it challenging to provide individual support, or even recognise that a child required additional support:

‘I have 120 children in the classroom. It’s supposed to be 60. It’s difficult to help learners individually. I see learners with these disabilities and I can see they are not understanding me, but I can’t stop to assist.’ (Teacher, mainstream school, Ntcheu)

High turnover rates of teachers were also observed, which could affect building rapport and understanding the educational needs of children with disabilities. The mother of a girl with a hearing impairment explained that ‘because the teachers keep changing, they don’t really get used to her condition, so it is hard for them to keep their attention on her’. She had gone to the school in the past to explain her daughter’s disability and ask for her to be placed at the front of the classroom to hear better, but was discouraged from continuing as ‘it is difficult because it means I need to be talking to every teacher that comes’.

The teachers interviewed reported receiving little to no training in inclusive education. One of the teachers at a mainstream school in Ntcheu, who had just finished her training, reflected on how inclusive education was covered:

‘[*T*]hey just said you would have children with disabilities in their class and to help them and treat them fairly, consider them. But we were just being warned that you have to make sure they understand.’ (Teacher, mainstream school, Ntcheu)

However, she and other mainstream school teachers reported that they were not provided with any specific teaching strategies or resources. Even teachers at resource schools appeared to have limited training. A teacher at a resource school in Ntcheu explained that she only taught children with more mild disabilities, whilst children with more severe disabilities never transitioned into the mainstream classes within the school. Similarly, a child with a profound hearing impairment who attended a resource school did not know formal sign language. The sibling of this child, who attended the same resource school, reported that the teachers did not use sign language with his brother but instead showed ‘interest’ and occasionally asked him to assist with communication using the informal signing method they had developed at home, which only allowed for limited, basic communication.

Physical accessibility of schools could also be challenging. Several mainstream and resource schools in both districts had built ramps to accommodate wheelchair users; however, some of these ramps would be difficult for children to use because of very steep inclines, disrepair, uneven and difficult terrain preceding ramps, and the presence of steps after ramps preventing the entry into classrooms and other facilities.

### Journey to school

A common barrier to attending schools was travel. Walking was the only mode of transport for almost all children. Almost half of children and caregivers reported challenges in getting to school, which was particularly common for children with mobility limitations. Difficulties in getting to school led to frequent absences and lost learning time for some children (boy, physical impairment, age 15, in school):

Boy ‘This year the school is close by; it is in the village. I could have been in Standard 8 or Form 1, but in the previous years it was difficult to get to school because the school is very far from the village. So now I am in standard 6.’

Interviewer ‘How far is the former school? If you start off at 6 AM, when do you get there?’

Boy ‘I could get there at around past 8 in the morning. I would find people already in class; sometimes I could get there at around past 9 … when I walk for a distance I need to sit down [*because of pain in my legs and back*], and my friends leave me behind.’

Distances to secondary schools were an anticipated challenge to transitioning, given the limited supply of schools. Similarly, travel to special and resource schools was an issue for the small number of children who had ever attended one of these institutions, as well as a perceived challenge that caregivers reported as a reason for not looking into these options. For example, a child with a profound hearing impairment had gone to a special school in another district for preprimary school. The fee for transport (Malawian kwacha 1600, approximately $6 per year) was cited by his caregiver as the main reason they had stopped sending him to the school. To reduce travel time for special and secondary schools, most children boarded either at or near the school, although this carried costs. Special or resource schools could start at preprimary, meaning that young children were away from their families for long periods of time. The caregiver of the boy with a hearing impairment who had gone to a boarding resource school as a young child noted safety concerns with boarding, such as that her child complained of not getting enough food, bullying by others and that the children were not properly supervised.

## Impact of non-inclusive education

### Poor learning outcomes

Although some children were doing well at schools, despite facing a range of challenges, the majority of participants reported that children with disabilities were performing poorly in terms of learning outcomes. Most children had repeated a grade, and many had repeated grades multiple times. This was attributed to children having missed time at schools because of illness or seeking healthcare or caregivers delaying enrolment, as well as due to a lack of effective inclusive education in schools. On average, children with disabilities were almost three grades behind the official national standard for their age. As a mother of a boy explained:

‘[*Being held back*] affects him very much. As you can see, he is very old compared to the class he is in. The friends that he started with are in Class 5; others are in Class 6, but he is just stuck in Class 2. This simply means that he is not able to hear what the teacher says, so I am always worried about my child when it comes to education, since it is not working properly and that’s bad for his life.’ (boy, aged 8, hearing impaired, in school)

Some children were promoted to higher grades even if they had not mastered the learning objectives for that level. The mother of a girl with a hearing impairment explained that she was upgraded to higher levels so that she would be with children closer to her own age, even though she did not pass her tests as ‘she is growing up and she can’t be remaining in the same class’.

### Social exclusion and isolation

Many children had difficulties keeping pace with the rest of their class and hence often repeated grades. Late starts and grade repetitions led to them being older than their classmates, thus contributing to feelings of low self-esteem amongst some children with disabilities. Reflecting on being held back a grade, one girl (aged 13, hearing impaired, in school) explained:

Interviewer ‘Why did you repeat?’

Girl ‘I was not intelligent enough … I didn’t know how to read and write.’

Interviewer ‘When you repeated, how did you feel about that?’

Girl ‘I felt bad because repeating a class means you’ve taken a step backwards in education.’

Interviewer ‘Did your other friends repeat too?’

Girl ‘No.’

Furthermore, as noted above, many children experienced bullying and stigmatisation by peers and teachers alike whilst attending the school. In some instances, these experiences affected children’s desire to attend school and caregivers’ willingness to send them. Even without overt discrimination, lack of accommodations in and outside the school could lead to social isolation. A 15-year-old girl with a visual impairment discussed how she was often either intentionally or unintentionally excluded from social activities with her peers:

‘Sometimes my friends run to school instead of walking, which makes it difficult for me because I can’t see properly. It makes me feel sad for myself … Sometimes they don’t want me to participate in the game, because they are worried they’ll hurt my eyes. That frustrates me.’ (girl, aged 15, visual impairment)

Decisions to drop out of school could have negative psychological impacts on children with disabilities. Children and their caregivers spoke of feelings of frustration and isolation after having to drop out. A 16-year-old girl who has a profound visual impairment discussed her desire to return to school, as she sits at home ‘admir[*ing*] my friends who go to school’. She reflected that not going to school ‘… hurts me because if I had continued to school, I would’ve been independent’. This concern about the impact on their future of not continuing with school, or of limited learning whilst in school, was repeated frequently by both children and their caregivers. The mother of a young man aged 18 with a hearing impairment who required frequent medical care for recurrent ear infections explained her son’s anguish when he had to drop out of secondary school after securing a coveted spot, because of the inability to afford school fees:

‘He [*wanted*] to do well in school so that he can have a bright future. He said for one to get a good job, you have to go to school … [*when he dropped out*] he cried the whole day. I also cried … I think [*now that he has dropped out*] his future will be difficult. Education is the only key to a successful future.’ (boy, aged 18, hearing impairment)

## Discussion

Overall, this research study found that children with disabilities in Mangochi and Ntcheu districts faced multiple barriers to participating and benefitting from education, which operated at the family, community, school and education system level. Disabling environments, including the lack of resources for inclusive education, inaccessible schools and teaching materials, inadequately trained teachers and negative attitudes on disability were major barriers preventing children with disabilities from attending and progressing in schools. These factors also affected their learning and social experiences at schools.

Understanding the barriers and enablers that affect access to education for children with disabilities in Malawi is essential for the country (as well as other LMICs) to meet international targets for universal education. This study reinforces and can help explain findings from quantitative research indicating that children with disabilities are less likely to attend and progress in education (Banks et al. 2017; Mizunoya et al. 2018; Simo Fotso et al. 2018; United Nations 2019). It also suggests that traditional metrics, such as attendance, attainment or grade level, may underestimate disparities in education between children with and without disabilities. This study and others have found that attending school or even progressing to higher grades may not be indicative of children with disabilities’ learning (Banks et al. 2019; Singal 2008). Whilst the quality of education is an issue for all children, children with disabilities appear more likely to be excluded from the learning process because of the lack of inclusive education provisions, including teacher training and specialist resources (Chitiyo et al. 2015; Jolley et al. 2018; Mkandawire, Maphale & Tseeke 2016; Taneja-Johansson et al. 2021). In the study areas, less than 2% of primary school classrooms had any inclusive education resources (MoEM 2021). The shortage of teachers trained in inclusive education is particularly high for secondary schools (Chitiyo et al. 2015).

Many barriers identified by this research affect access to education for all children, and not only children with disabilities. For example, poverty is the most common reason for school dropout nationally, and perceived costliness of fees is a major driver of failure to progress to secondary schools in the country for all children (MoEM 2021). Similarly, large class sizes affect all learners, as do frequent absences for work, poor health or other reasons. Still, children with disabilities are disproportionately affected by these challenges. For instance, extra costs associated with disability can exacerbate poverty and reduce capacity to pay for education (Mitra et al. 2017; Simeu & Mitra 2019). Other studies have found households incur additional direct and indirect costs related to sending children with disabilities to schools, such as for fees to specialist schools and resources, transportation or caregivers’ time accompanying children to school (Banks et al. 2021; Hanass-Hancock & McKenzie 2017; Kamaralzaman et al. 2018), although more research in this area is needed. The increased risk of poverty in households with a member with a disability affects the entire family, and it can have an impact on the educational outcomes of children without disabilities (Hailemichael et al. 2019; Simeu & Mitra 2019).

Similarly, large class sizes reduce individual attention for all learners. However, this study and others highlight that children with disabilities more likely need individualised supports – in some cases as minor as being moved to the front of the class – which teachers may not recognise when contending with class sizes of over 60 students (Mkandawire et al. 2016). More intensive support, such as instruction in sign language for children with profound hearing impairments or an adapted curriculum for children with intellectual impairments, was rarely provided to children in this study. Irregular attendance because of reasons such as the need to work, illness, inability to afford school fees and during menstruation has been observed for children without disabilities (Bodat, Ghate & Majumdar 2013; Weideman et al. 2007). However, children with disabilities in this and other studies also often had long and frequent absences and late starts to school, because of poor health conditions and the need for seeking health and rehabilitation services because of their impairment and underlying health conditions (Banks et al. 2017, 2019, 2021).

Another dominant theme through this research was the impact of negative attitudes and discrimination of disability. These attitudes could directly impact schooling decisions, such as when teachers requested or suggested children with disabilities not enrol or drop out, or when caregivers did not send their children to school with a belief that they could not benefit from an education. Bullying and mistreatment from peers and teachers and in the community could affect children’s desire to stay in school, their self-esteem and their social and learning experience whilst at school. The increased risk of children with disabilities – particularly girls with disabilities – to violence, including disability-targeted abuse and discrimination, has been observed in other studies (Banks 2017). In addition to the devastating toll on affected girls, the fear for children’s safety was a barrier to accessing education, as in most cases going to school implies the need to travel, sometimes of long distances.

Several limitations should be considered when interpreting the results of this study. Importantly, some children with severe intellectual impairments or profound hearing impairments with no sign language knowledge were not interviewed because of challenges in communication. In these cases, information was generally provided by the caregiver alone. Additionally, teacher interviews were not possible in Mangochi because of the start of the COVID-19 pandemic, meaning that the views of teachers in this study setting were not captured. There were also some differences in the study design between the two settings (e.g. age groups), which limit comparability. Finally, social desirability bias, or discomfort in disclosing sensitive information, may have led to underreporting on certain topics. However, interviews with caregivers, children and teachers brought multiple perspectives that allowed for the triangulation of data.

One of the valuable contributions of this research is that it shows how important it is to consult with children with disabilities directly about their own lives, rather than relying only on the input from caregivers and teachers. Certain topics that arose in the interviews, such as bullying, came out mostly from child interviews. Including meaningful participation of children with disabilities and promoting a rights-based approach in all education programmes and policies are essential to ensure that they are aligned with the UNCRPD and that children with disabilities are having their needs and rights met.

There are clear areas for further research, as well as some needing urgent attention from programmes and policymakers. There is a need to address the linkages between health, education and poverty amongst children with disabilities. This study has highlighted potential direct and indirect impacts of unmet health needs and poverty on education, indicating that health and social protection programmes are needed in addition to inclusive education initiatives. There may be a role to be played by multidimensional community-based rehabilitation programmes in coordinating responses between sectors. Overall, there is a lack of data on the effectiveness of interventions to improve access to education for children with disabilities in both Malawi and other LMICs (Jolley et al. 2018; Saran, White & Kuper 2020).

It is worth noting that the findings of this study echo those conducted in other LMICs (Magumise & Sefotho 2020; Okyere et al. 2019; Singal 2019; Singal et al. 2015) and some recent studies in Malawi (Phiri 2021). Challenges accessing education amongst children with disabilities are well-documented, as are difficulties implementing inclusive education in LMICs (De Souza 2021; Mphwina 2022; Sharma & Deppeler 2005; Van Tran et al. 2020). Whilst the present findings contribute to this broader literature, they also yield important insights for the Malawian context in particular.

As discussed in the Introduction section, there are three main options for education for children with disabilities in Malawi: education in mainstream schools without resource centres, education in mainstream schools with resource centres and education in specialised or segregated schools. There was a notable lack of discussion of resource centres by the participants, with most discussing challenges in inclusion in mainstream settings generally or barriers to accessing specialised or segregated schools. It is possible that these centres are not yet sufficiently widely available to be moving the needle on children with disabilities’ inclusion in schools. Increasing the number of schools that have the resources and facilities for inclusive education is a core aim of the SDG (Target 4a) and the UNCRPD. Coupled with the barriers and challenges associated with poverty, the apparent lack of accessible, meaningfully inclusive services for their children created sometimes insurmountable barriers to education.

Most notably, across the dataset, it was clear that the multidimensional poverty and the related barriers to education are primary drivers of low enrolment, attendance and attainment in schools for children with disabilities. It is likely that approaches to improve educational access and attendance in the country will need to include poverty-alleviation strategies which acknowledge the role of financial constraints and a range of deprivations in preventing children from achieving their full potential.

## Conclusion

Malawi has made strides in improving access to education for children with disabilities, as reflected in the increased attention to disability in its policies. However, this study indicates that further action is needed before inclusion in education can be a reality, particularly for poorer children and those with intellectual or communication disabilities.

Returning to the definition of inclusive education shared at the beginning of this article, it is possible to view challenges in all of its dimensions: in terms of the *longitudinal* aspects, it is clear that transitions to higher levels of education are extremely difficult to make, as is maintaining continual attendance during persistent health difficulties and the need for seeking health services; in terms of *location,* schools – particularly resource schools – are often geographically inaccessible to children; in terms of the *pedagogical* aspects of inclusion, there are material and human resource constraints on the quality of learning opportunities; and the *environmental* dimensions such as social inclusiveness and physical accessibility are lagging; all of which result in the *consequences* of education – educational inclusion, participation and attainment – being stymied.

There is a need for investing in support for families, schools and communities, as well as in laws, policies and monitoring mechanisms, so that positive, inclusive school experiences for children with disabilities are not only possible but also the norm. The commitments in the SDGs and the UNCRPD to an ‘inclusive and equitable quality education’ will not be met unless there is increased investment and prioritisation not only of inclusive education but also to broader disability-inclusive planning (e.g. in health systems, social protections and poverty alleviation programmes).

## Footnotes

1 Some of the data reported in this study come from a non-peer reviewed report produced at the project endline (Banks & Zuurmond 2015). This article significantly expands upon those findings.
